# Integrative anatomical and two-dimensional ultrasonographic assessment of the heart in Shetland ponies

**DOI:** 10.3389/fvets.2025.1721000

**Published:** 2026-01-15

**Authors:** Jorge Isidoro Matos, Lidia Pitti, María Parra-Quijano, Alberto Arencibia, Gregorio Ramírez, María Luisa Díaz-Bertrana

**Affiliations:** 1 Facultad de Medicina Veterinaria, Instituto de Investigación en Ciencias Biomédicas y de la Salud (IUIBS), Universidad de Las Palmas de Gran Canaria, Las Palmas de Gran Canaria, Spain; 2 Facultad de Veterinaria, Hospital Clínico Veterinario, Universidad de Las Palmas de Gran Canaria, Las Palmas de Gran Canaria, Spain; 3 Facultad de Veterinaria, Hospital Clínico Veterinario, Universidad de Murcia, Murcia, Spain; 4 Facultad de Veterinaria, Departamento de Morfología, Universidad de Las Palmas de Gran Canaria, Las Palmas de Gran Canaria, Spain; 5 Departamento de Anatomía y Anatomía Patológica, Universidad de Murcia, Murcia, Spain

**Keywords:** anatomy, dissection, echocardiography, equine, heart, Shetland pony, ultrasound, veterinary

## Abstract

**Objective:**

The purpose of this study was to characterize the normal anatomy of the Shetland pony heart through an integrative approach combining two-dimensional transthoracic echocardiography and anatomical dissection.

**Methods:**

Transthoracic echocardiographic examinations were performed in 19 clinically healthy Shetland ponies, obtaining standardized longitudinal and cross-sectional two-dimensional views of the heart. These images were used to describe the main echocardiographic windows and to document the visualization of relevant cardiac structures. In parallel, four Shetland pony hearts were dissected and sectioned, allowing an illustrative correspondence between anatomical landmarks and representative echocardiographic views, thereby facilitating a more precise interpretation of the observed structures. Furthermore, basic two-dimensional echocardiographic measurements were determined, including diameters of key cardiovascular chambers and vessels.

**Results:**

Nineteen Shetland ponies (median age 6.8 years; median body weight 136.9 kg) were evaluated. Anatomical analysis allowed identification of the main cardiac structures and their topographic relationships, which showed illustrative correspondence with the two-dimensional echocardiographic views. The largest measurements were the left ventricular diastolic diameter (5.4 cm) and the left atrial diastolic diameter (4.2 cm), while the smallest was the right ventricular free wall in diastole (1.2 cm). Intra-observer variability was low (ICC = 0.94; CV = 3.2%), confirming high measurement repeatability.

**Conclusion:**

Overall, the data obtained provide an adequate anatomical reference and offer relevant insights to support the interpretation of transthoracic two-dimensional echocardiographic studies in Shetland ponies.

## 1 Introduction

The Shetland pony is one of the smallest equine breeds, originating from the Shetland Islands in northern Scotland and considered a modern native pony of Great Britain (1). Adults typically measure 90–110 cm at the withers and weigh between 100 and 300 kg. Despite their reduced stature, Shetland ponies are notably robust, with compact bodies, short but strong limbs, and dense coats well adapted to harsh climates (2, 3).

The morphological characteristics of the Shetland pony have attracted considerable interest, particularly the anatomical adaptations associated with its markedly reduced body size (4, 5). This breed is characterized by a proportionally large head, relatively short but sturdy limbs, and broad thoracic and abdominal cavities that accommodate well-developed organ systems (6, 7). Anatomical differences are especially evident in the skull, where paranasal sinuses and sinonasal communications differ substantially from those of standard-sized horses (8). Cranial proportions are also distinctive, with Shetland ponies exhibiting a comparatively larger cranium and smaller muzzle than larger horse breeds and donkeys (9). The vertebral column shows a particular configuration, with a greater tendency toward thoracoization and lumbarization, together with increased variability in the number of vertebrae and rib pairs. Furthermore, the limb skeleton is formed by relatively short yet compact and resistant bones, contributing to the strength and endurance that distinguish the breed in relation to its body size (10).

From a cardiovascular perspective, ponies, including Shetlands, generally present relatively smaller cardiac chambers but proportionally thicker ventricular walls compared to larger horses, consistent with their compact conformation and higher relative cardiac workload (7, 11). Echocardiography has been validated in this breed, and several studies have highlighted important breed-specific cardiovascular features. For example, Shetland ponies with metabolic disorders exhibit increased echocardiographic values of left ventricular wall thickness compared to healthy standard horses (11, 12). Non-uniform patterns of blood pressure and heart rate regulation have also been reported, alongside associations between heightened sympathetic tone, reduced parasympathetic tone, and ventricular wall thickening (13). These findings suggest that Shetland ponies may exhibit slightly elevated blood pressure, predisposing them to left ventricular hypertrophy, a condition strongly linked to the high prevalence of obesity within this breed (14, 15). Furthermore, congenital cardiovascular defects have been documented, including ventricular septal defects, absence of the pulmonary valve, and anomalous pulmonary artery development, as confirmed through echocardiography and post-mortem anatomical dissection (16).

In addition, a recent study in horses and ponies of various breeds reported echocardiographic reference values using allometric regression equations based on body weight and thoracic circumference. These results demonstrated a direct relationship between echocardiographic parameters and body size, underscoring the importance of breed-specific reference data to improve the diagnostic accuracy and prognosis of equine heart disease (17).

Although functional cardiovascular adaptations in Shetland ponies have been documented, this study was intentionally limited to structural assessment. Functional echocardiographic parameters, such as systolic or diastolic function indices, were excluded due to their reliance on advanced imaging techniques and specific hemodynamic measurements. The primary aim was to establish a detailed anatomical and echocardiographic reference based on two-dimensional views. Given the breed’s unique cardiovascular morphology, using reference values from larger horse breeds may lead to diagnostic inaccuracies. Breed-specific data are therefore essential to ensure reliable echocardiographic assessment.

Previous studies have typically addressed either anatomical or echocardiographic aspects in isolation, with limited efforts to visually correlate both modalities. This study addresses that gap by providing a combined anatomical–echocardiographic reference specific to the Shetland pony, without subject-specific matching. This approach is intended to support both clinical interpretation and veterinary education by offering illustrative visual comparisons between anatomical structures and standard echocardiographic views.

## 2 Materials and methods

### 2.1 Animals subjected to echocardiography

This was an exploratory, descriptive study aimed at generating baseline anatomical and echocardiographic reference information for the Shetland pony. A prospective study was conducted between September 2023 and July 2025, involving a total of 19 Shetland ponies from the island of Gran Canaria (Spain). Various descriptive parameters were recorded for each animal, including age (years and months), body weight (kg), body condition score (BCS: 1–9) (18), reproductive status (sterilized or not), heart rate (beats per minute), and height at the withers (cm). Weight was estimated using costal arch circumference (cm) and total body length (cm) following the formula: Weight = (CA^2^ × TL) / 1900, in accordance with previously established protocols (19). Additional data collected included geographic location, history of previous illnesses, and any medical treatments administered. Echocardiographic examinations were performed at the animals’ place of habitation without relocating them for the procedure.

No formal sample size calculation was performed, as the number of animals included was based on availability and adherence to the inclusion criteria. Animals were included in the study if they met the following criteria: Pure Shetland breed, body weight under 300 kg, minimum age of 1 year, withers height below 110 cm, no history of cardiopulmonary disease, no current pharmacological treatment, no sedation or anesthesia during the ultrasound, compliance with regional regulations on deworming, vaccination, and animal identification, as established by the Autonomous Community of the Canary Islands (Spain). To further ensure the health status of the animals, all individuals underwent a targeted physical examination performed by veterinarians with extensive expertise in equine medicine (M. L. D.-B., L. P.). The clinical evaluation included assessment of heart rate, respiratory rate, thoracic auscultation, mucous membrane color, and capillary refill time. Additionally, all animals underwent transthoracic two-dimensional echocardiographic screening. Only ponies with no relevant clinical signs and no detectable structural or functional cardiac abnormalities were included in the study.

### 2.2 Echocardiographic evaluation

All 19 ponies underwent a standardized transthoracic echocardiographic examination. Static images and cine loops were acquired in two-dimensional mode using the same ultrasound system and a 1.2–4.5 MHz phased-array transducer (Vivid iq, General Electric, Boston, MA, USA). Ponies were examined while standing in a relaxed position. Standardized longitudinal and transverse right and left parasternal views were acquired following the protocol adapted for equine described by Koenig et al. (20). For the right parasternal approach, the transducer was positioned between the third and fourth intercostal spaces to obtain standard right-sided long- and short-axis views. For the left apical approach, the most caudoventral region of the left hemithorax was explored, specifically between the fifth and sixth intercostal spaces, as close as possible to the cardiac apex, which allowed acquisition of left-sided views in all animals.

The right-sided transverse sections included views at the levels of the: left ventricle, mitral valve, cardiac base, pulmonary trunk bifurcation. The right-sided longitudinal sections included: four-chamber view and five-chamber view. The left-sided longitudinal views included: apical four-chamber view and apical five-chamber view. All examinations were conducted with animals fully conscious, without anesthetic or sedative administration. For each parameter, three consecutive cardiac cycles in sinus rhythm were recorded, obtaining the arithmetic mean. All echocardiographic evaluations were performed by the same investigator (J. I. M).

The following two-dimensional measurements were obtained following established protocols (17): Interventricular septum diameter in systole and diastole (IVSs, IVSd) from a right longitudinal four-chamber view, Left atrial diameter in systole and diastole (LAs, LAd) from a right transverse view at the cardiac base, Right atrial diameter in systole and diastole (RAs, RAd) from a left longitudinal four-chamber view, Left ventricular cavity diameter in systole and diastole (LVs, LVd) from a right transverse view at the left ventricle level, Right ventricular cavity diameter in systole and diastole (RVs, RVd) from a left longitudinal four-chamber view, Left ventricular free wall thickness in systole and diastole (LVFWs, LVFWd) from a right longitudinal four-chamber view, Right ventricular free wall thickness in systole and diastole (RVFWs, RVFWd) from a right longitudinal four-chamber view, Tricuspid and mitral valve annular diameters (TVD, MVD) from a left longitudinal four-chamber view, Pulmonary and aortic valve diameters (PVD, AoVD) from a right transverse view at the level of the pulmonary trunk bifurcation.

In the absence of electrocardiographic tracing, end-diastolic and end-systolic frames were identified using consistent echocardiographic landmarks. When the atrioventricular valves (mitral or tricuspid) were visible, end-diastole was defined as the frame just before valve closure, corresponding to maximal ventricular internal dimension. In views where these valves were not present, end-diastole was identified as the frame immediately before aortic or pulmonary valve opening. End-systole was defined as the frame corresponding to minimal left ventricular cavity size, typically following semilunar valve closure.

### 2.3 Anatomical study

A total of 4 frozen cardiac samples from Shetland pony specimens were available for cardiac dissection and macroscopic analysis. Dissections were carried out at the Department of Veterinary Anatomy and Embryology (Faculty of Veterinary Medicine, University of Murcia). The four cardiac samples analyzed did not correspond to any of the 19 animals subjected to echocardiography. Specifically, the anatomical samples were obtained from animals that were euthanized due to traumatic and/or joint lesions that prevented an acceptable quality of life. All dissected animals were adults with ages and body weights comparable to those of the echocardiographic group. This comparison was made descriptively, without formal statistical analysis, due to the limited number of anatomical specimens.

**Figure 1:**
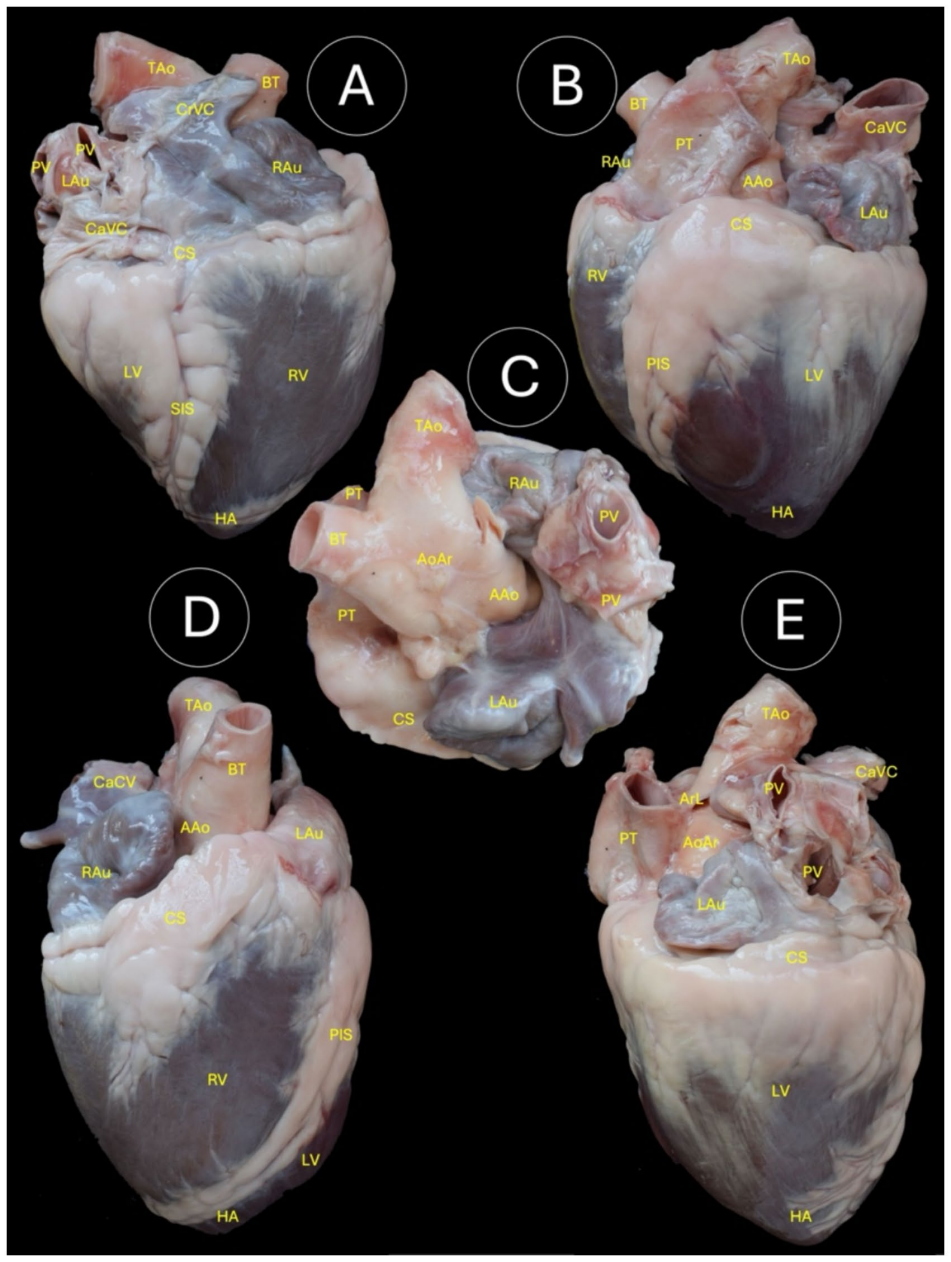
Anatomical dissections of the heart of the Shetland pony. **(A)** Atrial surface; **(B)** Auricular surface; **(C)** Dorsal surface; **(D)** Cranial view; **(E)** Caudal view. TAo (Thoracic aorta), CrVC (Cranial vena cava), BT (Brachiocephalic trunk), PV (Pulmonary veins), LAu (Left auricle), CaVC (Caudal vena cava), CS (Coronary sulcus), RAu (Right auricle), LV (Left ventricle), RV (Right ventricle), SIS (Subsinuosal interventricular sulcus), HA (Heart apex), PT (Pulmonary trunk), AAo (Ascending aorta), PIS (Paraconal interventricular sulcus), AoAr (Aortic arch), and ArL (Arteriosus ligament).

Anatomical reference texts (21, 22), in addition to previous dissection protocols (23), were consulted to guide macroscopic measurements and anatomical sectioning. External cardiovascular morphology was assessed, including the: Atrial surface, Auricular surface, dorsal surface, cranial and caudal views (Figure 1).

The anatomical preparations were thawed and dissected without using any chemical fixatives or preservation agents. This anatomical samples did not correspond to the individuals examined by echocardiography, therefore, comparisons were conceptual. Transverse sections were performed to replicate echocardiographic cross-sections at the levels of: Pulmonary artery bifurcation, cardiac base, mitral valve and left ventricle. Longitudinal anatomical sections were also obtained with and without including the left ventricular outflow tract and the aortic valve.

### 2.4 Statistical analysis

All continuous variables were assessed for normality using the Shapiro–Wilk test. Since all parameters showed non-normal distribution, results are expressed as median (minimum–maximum). Intra-observer reproducibility was determined from two independent measurements per animal. Intraclass correlation coefficients (ICC) were calculated, together with their 95% confidence intervals. Additionally, Bland–Altman plots were generated to estimate the mean bias and limits of agreement (mean difference ± 1.96·SD). As complementary indices of measurement error, the intra-observer coefficient of variation (CV%) was also calculated. All statistical analyses were performed using IBM SPSS Statistics, version 25 (IBM Corp., Armonk, NY, USA), with the level of significance set at *p* < 0.05.

### 2.5 Ethical considerations

All animal owners were informed and provided signed consent for participation in the study. This included authorization for the echocardiographic examinations as well as for the use of cardiac samples obtained post-mortem from animals subjected to necropsy. The study was observational and non-invasive, and its evaluation included ethical considerations and legal aspects regarding animal protection and welfare, carried out in accordance with current Spanish and European legislation on animal protection.

## 3 Results

**Figure 2:**
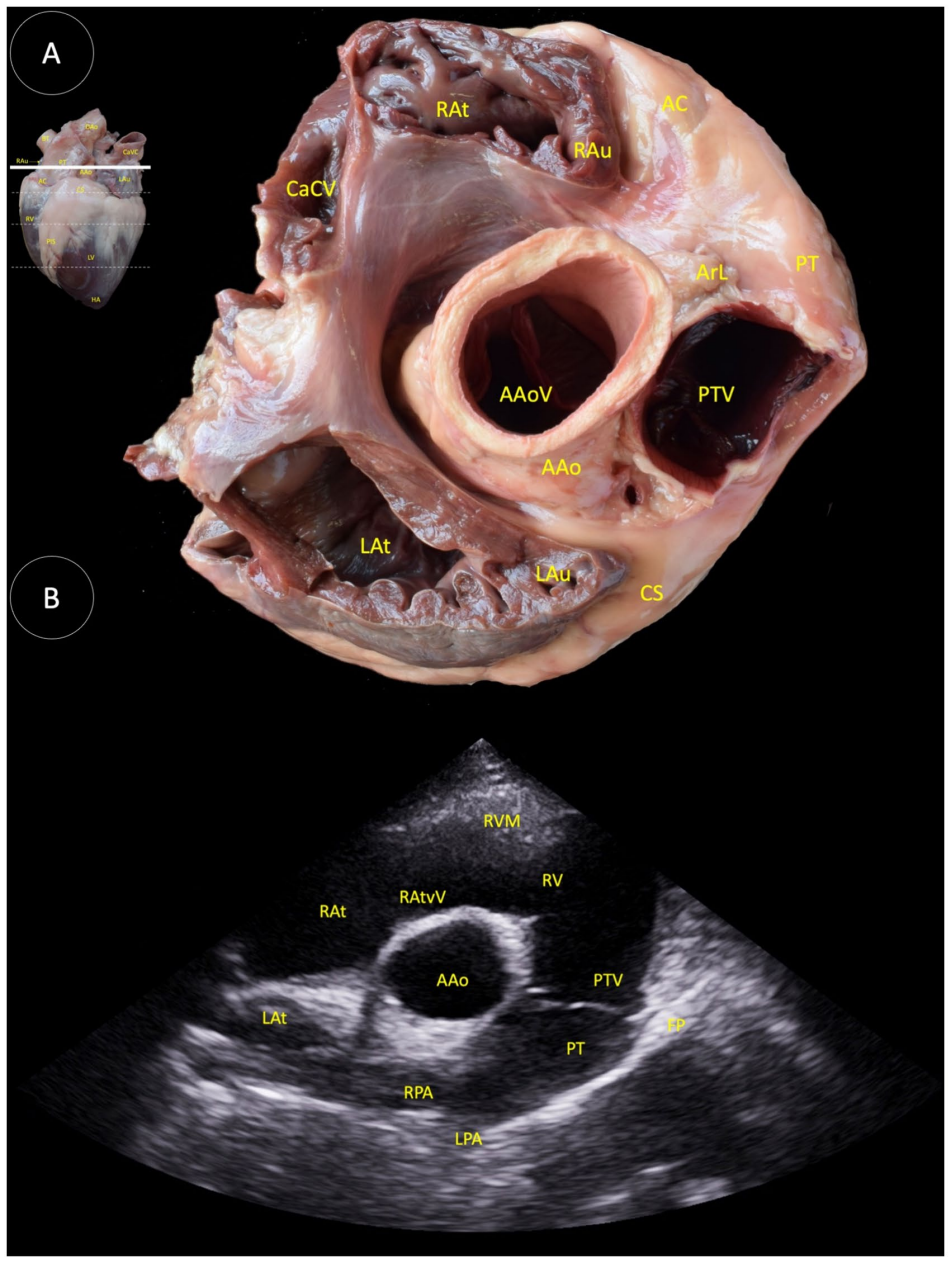
**(A)** Dorsal view of a transverse anatomical section of the heart of the Shetland pony at the right and left atrial level, illustrating the structures observed in the corresponding echocardiographic view. **(B)** Two-dimensional image for the right parasternal access, transverse view optimized for the view of the pulmonary trunk bifurcation. RAt (Right atrium), RAu (Right auricle), AC (Angular cuspid), CaCV (Caudal vena cava), ArL (Arteriosus ligament), PT (Pulmonary trunk), PTV (Pulmonary trunk valve), AoV (Aortic valve), AAo (Ascending aorta), LAt (Left atrium), LAu (Left auricle), CS (Coronary sulcus), RVM (Right ventricular muscle), RV (Right ventricle), RAtvV (Right atrioventricular valve), RPA (Right pulmonary artery), LPA (Left pulmonary artery), and TP (Tricuspid papillary).

**Figure 3:**
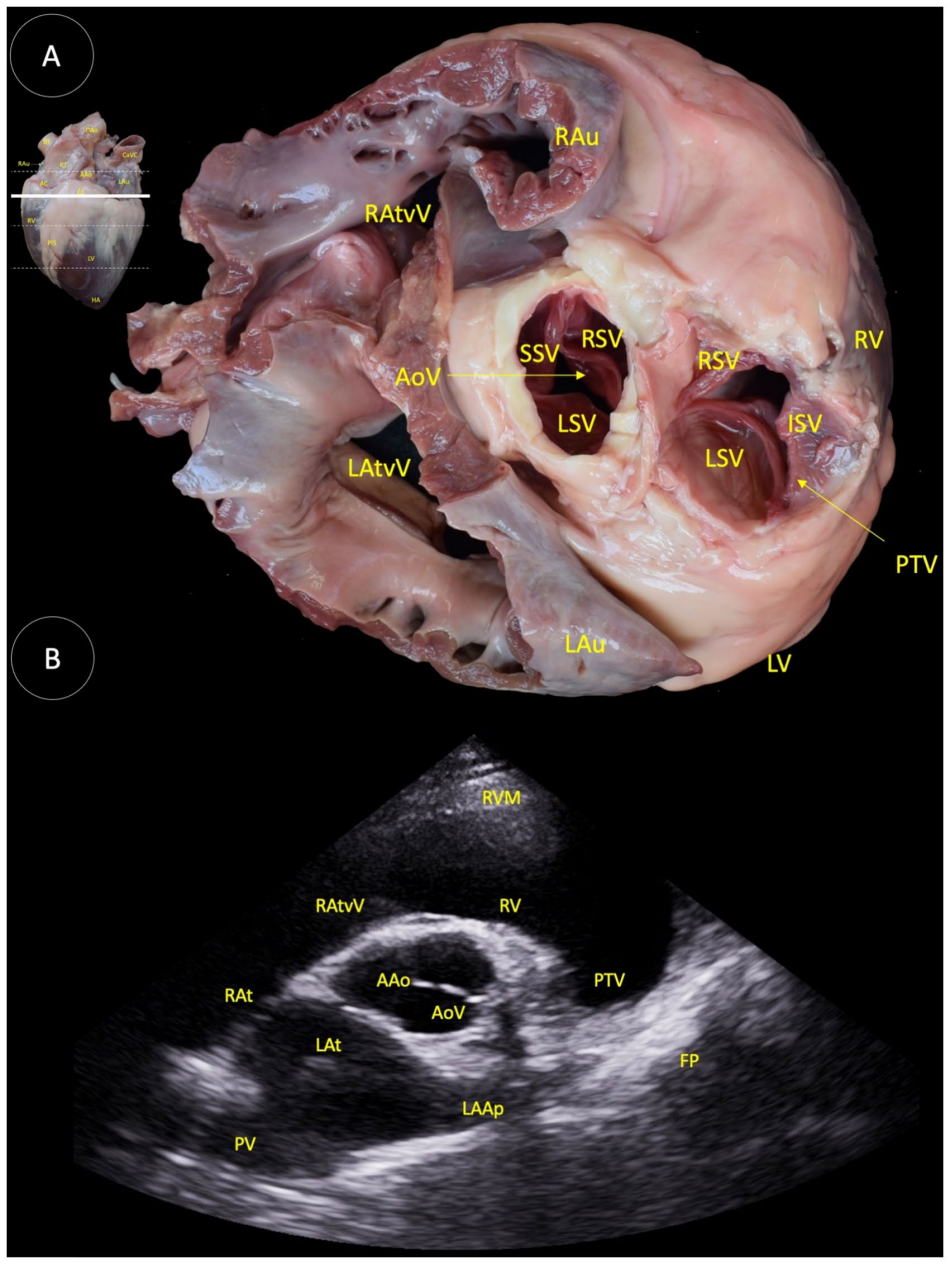
**(A)** Dorsal view of a transverse anatomical section of the heart of the Shetland pony at the aortic valve level, illustrating the structures observed in the corresponding echocardiographic view. **(B)** Two-dimensional image for the right parasternal access, transverse view at the heart base level. RAu (Right auricle), RAtvV (Right atrioventricular valve), AoV (Aortic valve), LAtvV (Left atrioventricular valve), LAu (Left auricle), RSV (Right semilunar valvule), SSV (Septal semilunar valvule), LSV (Left semilunar valve), PTV (Pulmonary trunk valve), RV (Right ventricle), LV (Left ventricle), AAo (Ascending aorta), RVM (Right ventricular muscle), RAt (Right atrium), LAt (Left atrium), PV (Pulmonary veins), LAAp (Left atrial appendage), and FP (Fibrous pericardium).

**Figure 4:**
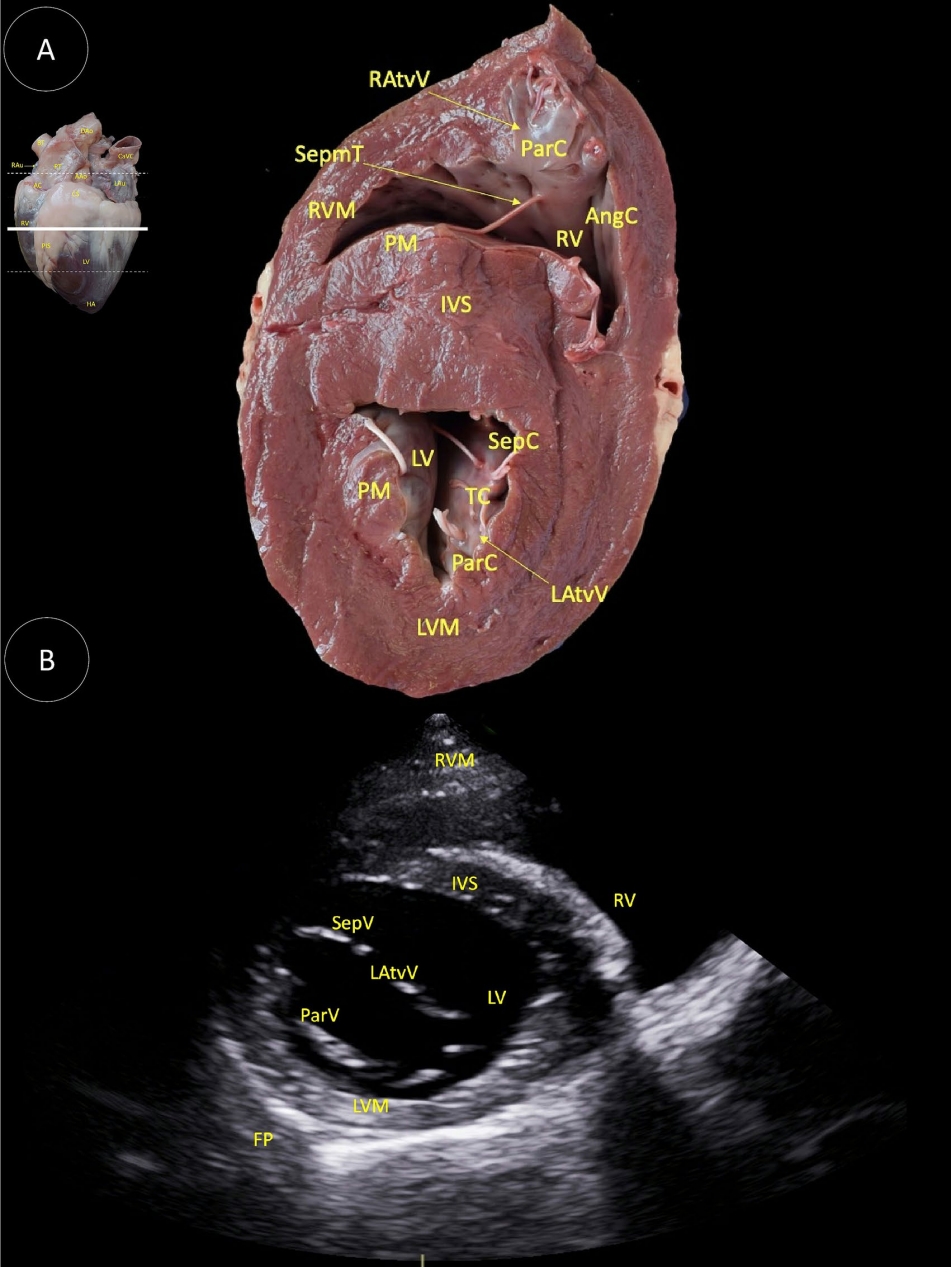
**(A)** Dorsal view of a transverse anatomical section of the heart of the Shetland pony at the mitral valve level, illustrating the structures observed in the corresponding echocardiographic view. **(B)** Two-dimensional image for the right parasternal access, transverse view at the mitral valve level. RAtvV (Right atrioventricular valve), SepmT (Septomarginal trabecula), RVM (Right ventricular muscle), PM (Papillary muscle), IVS (Interventricular septum), ParC (Parietal cuspid), AngC (Angular cuspid), RV (Right ventricle), LV (Left ventricle), SepC (Septal cuspid), TC (Tendinous cord), LAtvV (Left atrioventricular valve), LVM (Left ventricular muscle), SepV (Septal valvule), ParV (Parietal valvule), and FP (Fibrous pericardium).

**Figure 5:**
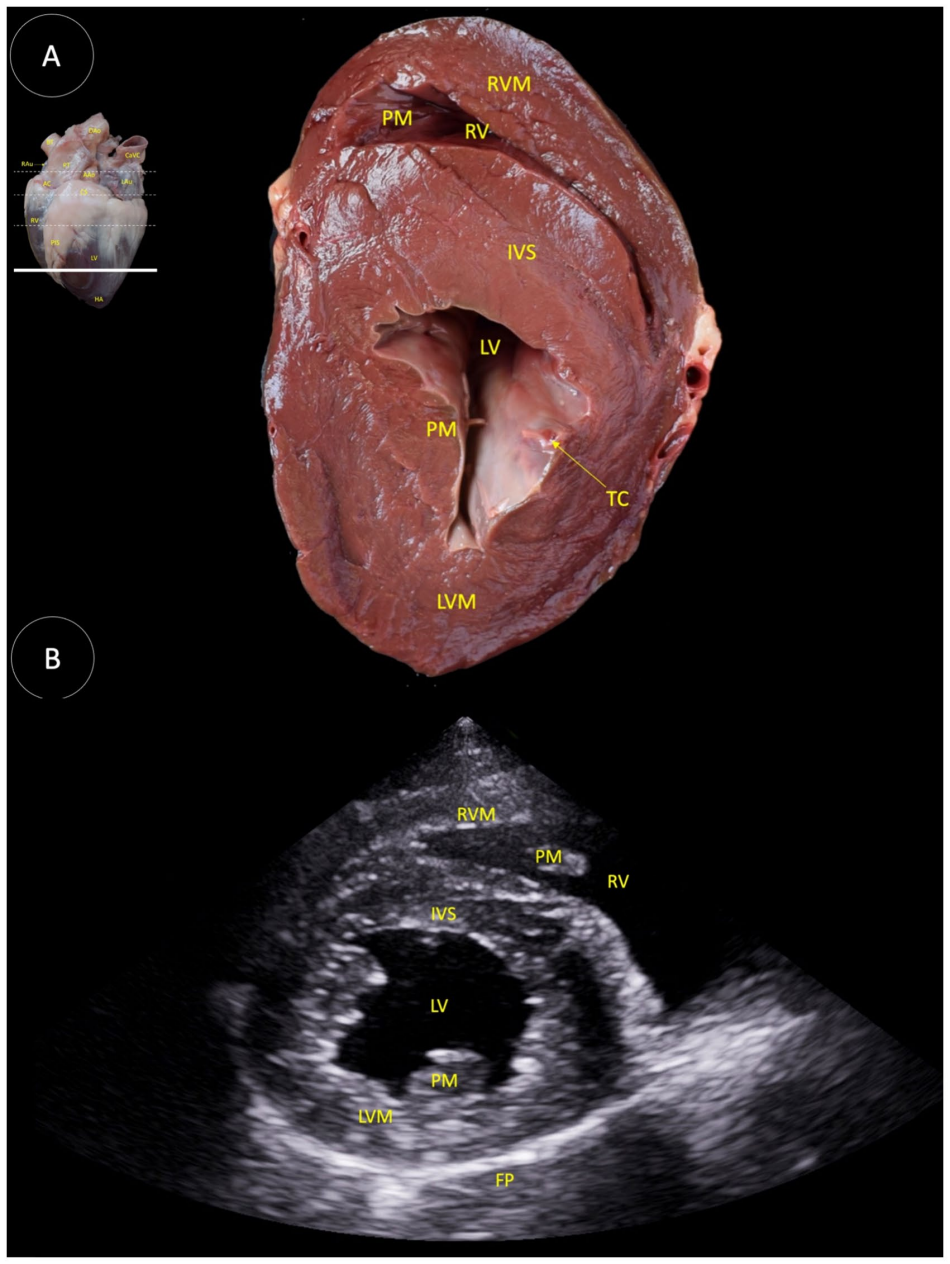
**(A)** Dorsal view of a transverse anatomical section of the heart of the Shetland pony at the papillary muscles level, illustrating the structures observed in the corresponding echocardiographic view. **(B)** Two-dimensional image for the right parasternal access, short axis view at the left papillary muscles level. RVM (Right ventricular muscle), PM (Papillary muscle), RV (Right ventricle), IVS (interventricular septum), LV (Left ventricle), TC (Tendinous cord), LVM (Left ventricular muscle), and FP (Fibrous pericardium).

**Figure 6:**
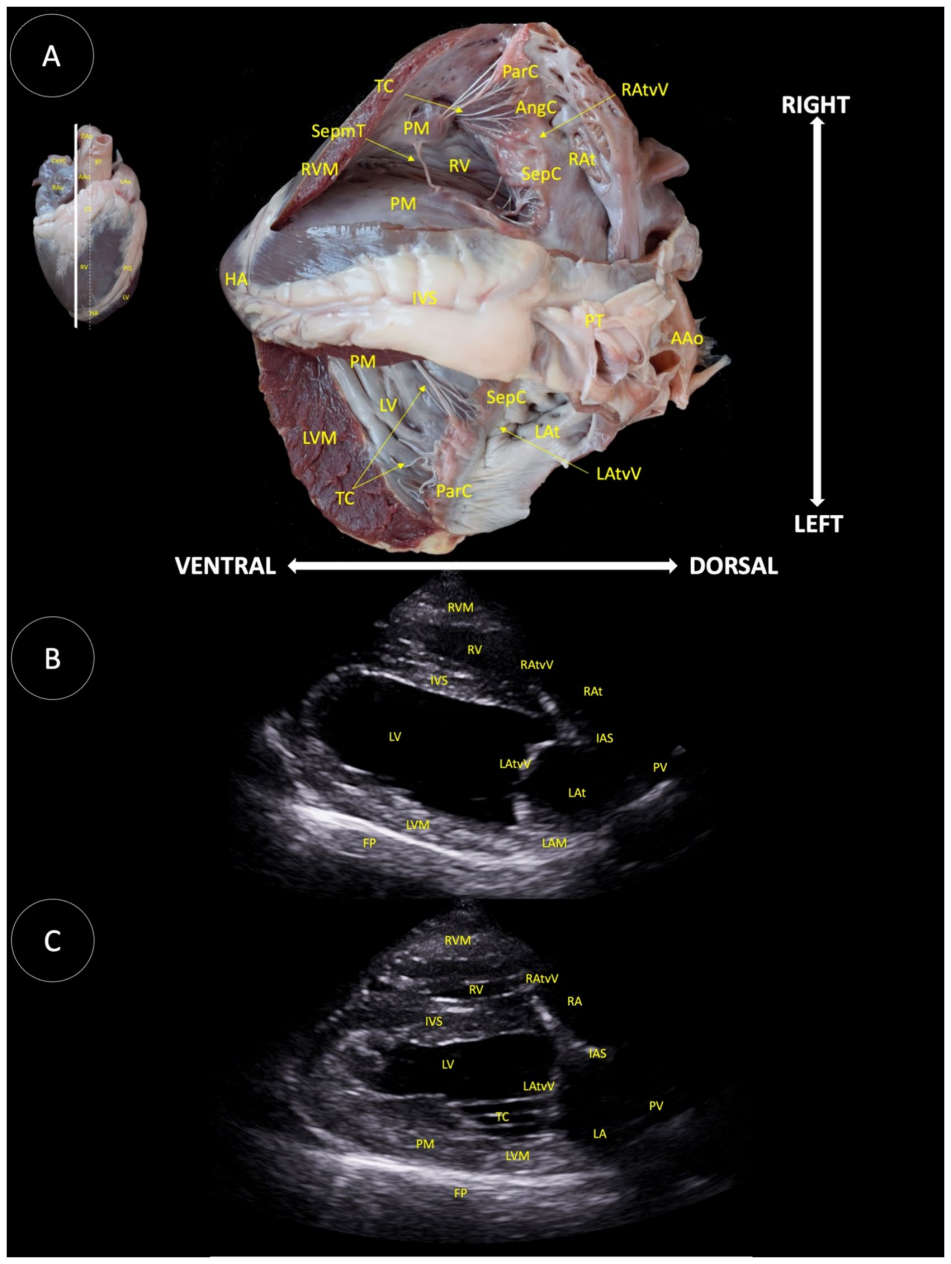
**(A)** Longitudinal anatomical section of the heart of the Shetland pony, illustrating the structures observed in the corresponding echocardiographic views. **(B)** Two-dimensional image for the right parasternal longitudinal 4-chamber view by the time of systole. **(C)** Two-dimensional image for the right parasternal longitudinal 4-chamber view by the time of diastole. TC (Tendinous cord), ParC (Parietal cuspid), AngC (Angular cuspid), RAtvV (Right atrioventricular valve), SepmT (Septomarginal trabecula), PM (Papillary muscle), RVM (Right ventricular muscle), RV (Right ventricle), SepC (Septal cuspid), RA (Right atrium), HA (Heart apex), IVS (Interventricular septum), RM (Right marginal), LV (Left ventricle), LVM (Left ventricular muscle), LAt (Left atrium), AAo (Ascending aorta), PT (Pulmonary trunk), LAtvV (Left atrioventricular valve), FP (Fibrous pericardium), IAS (Interatrial septum), PV (Pulmonary veins), and LA (Left atrium).

**Figure 7:**
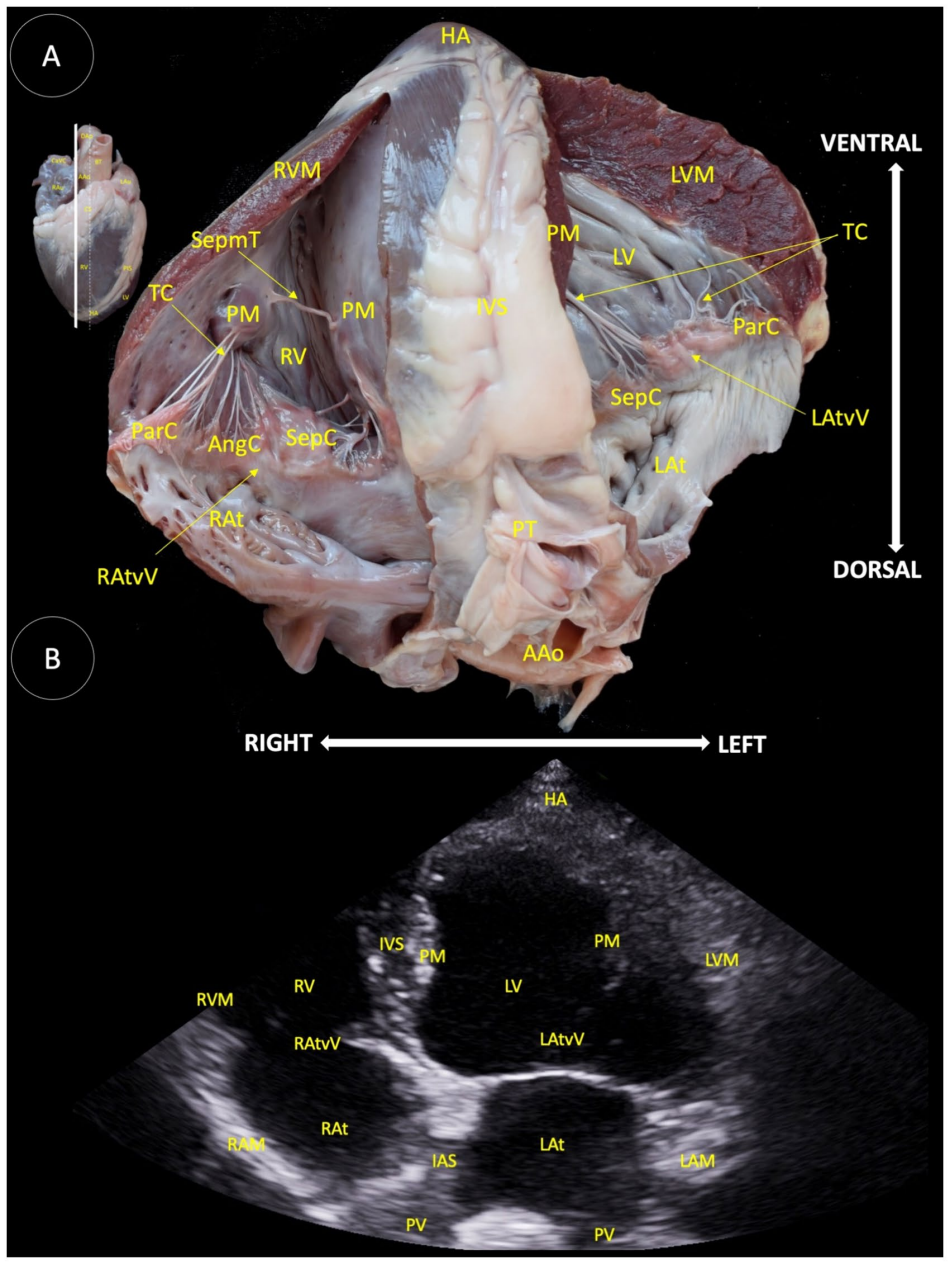
**(A)** Longitudinal anatomical section of the heart of the Shetland pony, illustrating the structures observed in the corresponding echocardiographic view. **(B)** Two-dimensional image for the longitudinal left parasternal access, four chamber apical view. HA (Heart apex), RVM (Right ventricular muscle), SepmT (Septomarginal trabecula), TC (Tendinous cord), PM (Papillary muscle), RV (Right ventricle), ParC (Parietal cuspid), AngC (Angular cuspid), RA (Right atrium), RAtvV (Right atrioventricular valve), SepC (Septal cuspid), IVS (Interventricular septum), LV (Left ventricle), LVM (Left ventricular muscle), LAtvV (Left atrioventricular valve), LAt (Left atrium), pulmonary trunk (PT), ascending aorta (AAo), IAS (Interatrial septum), PV (Pulmonary veins), and LAM (Left atrium muscle).

**Figure 8:**
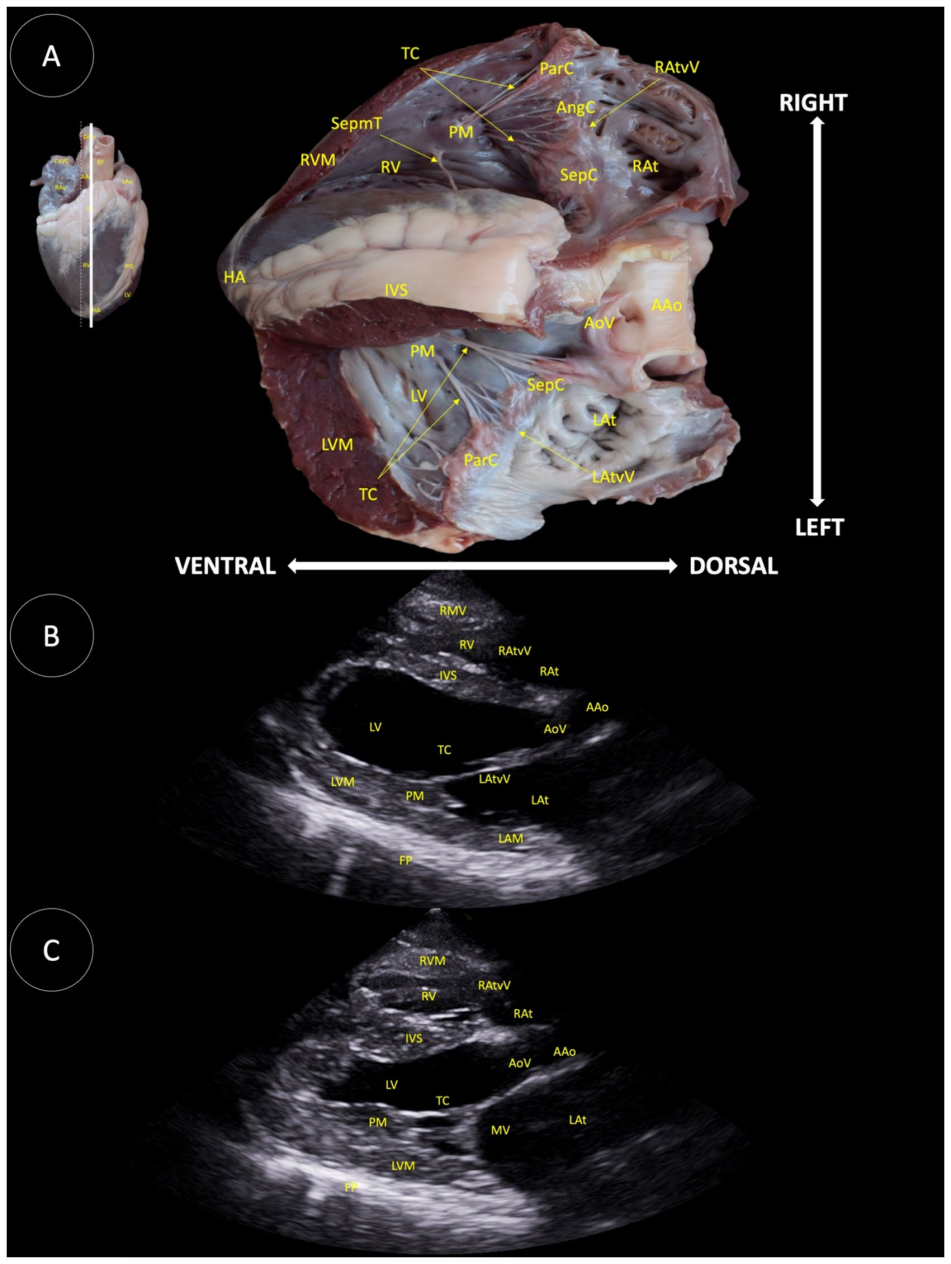
**(A)** Longitudinal anatomical section of the heart of the Shetland pony including the left ventricular outflow tract and the aortic valve, illustrating the structures observed in the corresponding echocardiographic views. **(B)** Two-dimensional image for the right parasternal longitudinal 5-chamber view by the time of systole. **(C)** Two-dimensional image for the right parasternal longitudinal 5-chamber view by the time of diastole. HA (Heart apex), RVM (Right ventricular muscle), SepmT (Septomarginal trabecula), TC (Tendinous cord), PM (Papillary muscle), RV (Right ventricle), ParC (Parietal cuspid), AngC (Angular cuspid), RA (Right atrium), RAtvV (Right atrioventricular valve), SepC (Septal cuspid), IVS (Interventricular septum), LV (Left ventricle), LVM (Left ventricular muscle), LAtvV (Left atrioventricular valve), LAt (Left atrium), AoV (Aortic valve), AAo (Ascending aorta), FP (Frontal plane), LAM (Left atrium muscle), and MA (Mitral valve).

**Figure 9:**
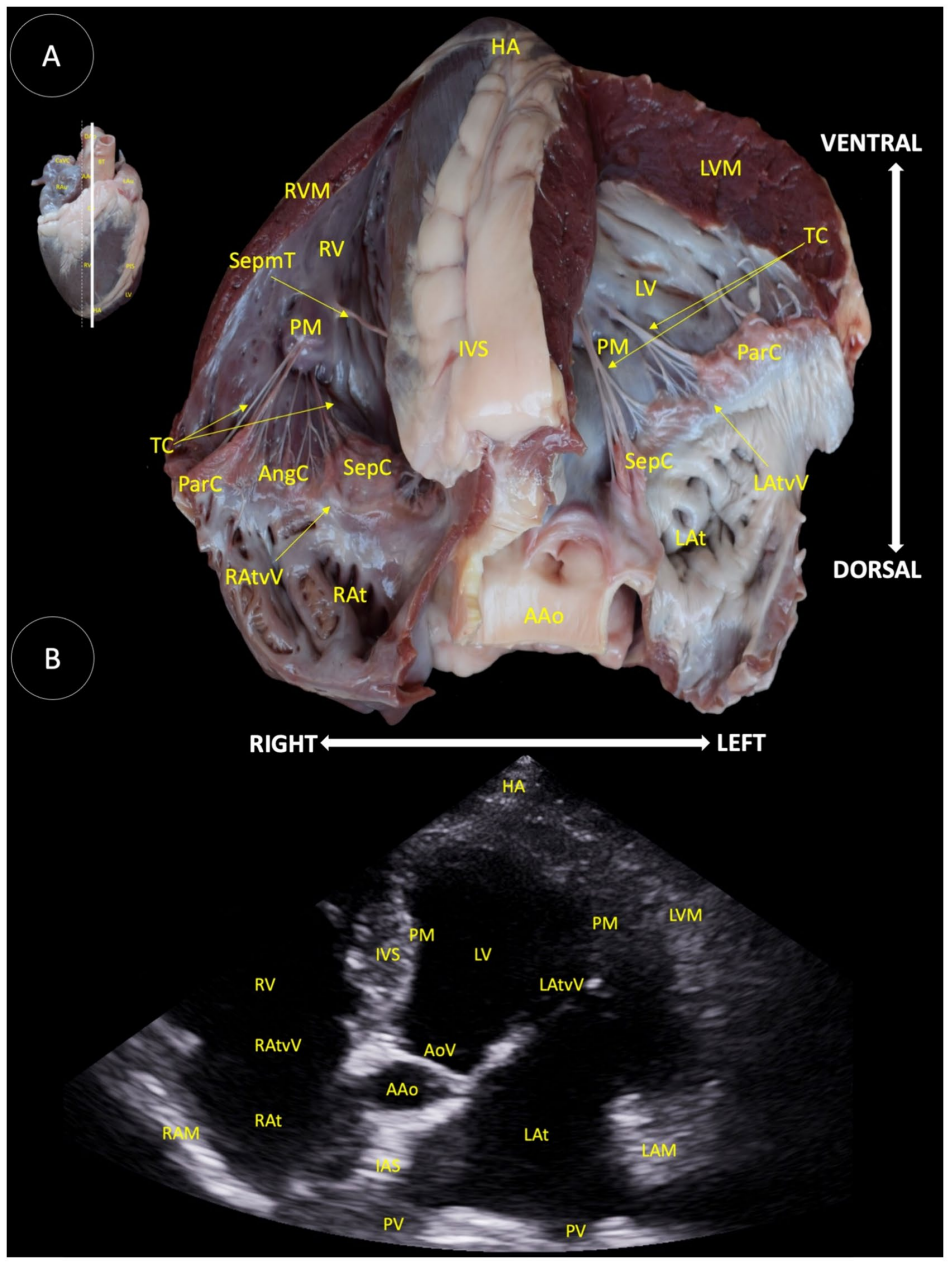
**(A)** Longitudinal anatomical section of the heart of the Shetland pony including the left ventricular outflow tract and the aortic valve, illustrating the structures observed in the corresponding echocardiographic view. **(B)** Two-dimensional image for the longitudinal left parasternal access, five chamber apical view. The heart apex (HA), right ventricle muscle (RVM), septomarginal trabecula (SepmT), right ventricle (RV), papillary muscle (PM), interventricular septum (IVS), tendinous cord (TC), parietal cuspid (ParC), angular cuspid (AngC), septal cuspid (SepC), right atrioventricular valve (RAtvV), right atrium (RAt), ascending aorta (AAo), left ventricle (LV), left atrium (LAt), left atrioventricular valve (LAtvV), left ventricle muscle (LVM), fibrous pericardium (FP), aortic valve (AoV), pulmonary veins (PV), interventricular septum (IAS), and left auricle mean diameter (LAM).

Table 1 shows descriptive data from the group of 19 individuals, including 4 intact males, 4 castrated males, and 11 females. The data are presented as median, minimum, and maximum, or percentages for the different variables collected. Likewise, the external anatomical structures and their topographic location in Shetland pony hearts are shown in Figure 1. On the other hand, Figures 2–9 show the echocardiographic views and their respective reference anatomical images.

**Table 1:** Representation of the most important clinical findings of the Shetland ponies analyzed (*N* = 19).

Descriptive parameters	Value (N = 19)
Age (years)	6.8 (3.0–11.2)
Female: number (%)	11 (57.9%)
Neutered males: number (%)	4 (50.0%)
Intact males: number (%)	4 (50.0%)
Body weight (Kg)	136.9 (80.1–208.2)
Costal arch (cm)	115.1 (92.9–149.3)
Total length (cm)	90.4 (72.0–118.3)
Height at withers (cm)	89.2 (83.1–104.6)
Body score condition (1–9)	6.2 (5.1–8.0)
Heart rate (beats per minute)	48.4 (35.3–58.5)

The categorical variables are represented numerically and the percentage. Quantitative variables are represented by median, minimum, and maximum.

The external morphological analysis enabled the study of the main anatomical structures, and was shown in Figure 1. In the atrial surface (view A), the thoracic aorta (TAo), cranial vena cava (CrVC), brachiocephalic trunk (BT), pulmonary veins (PV), left auricle (LAu), caudal vena cava (CaVC), coronary sulcus (CS), right auricle (RAu), left ventricle (LV), right ventricle (RV), subsinuosal interventricular sulcus (SIS), and heart apex (HA) were observed. In the auricular surface (view B), the brachiocephalic trunk (BT), thoracic aorta (TAo), pulmonary trunk (PT), ascending aorta (AAo), caudal vena cava (CaVC), left auricle (LAu), coronary sulcus (CS), angular cuspid (AC), right auricle (RAu), left ventricle (LV), right ventricle (RV), paraconal interventricular sulcus (PIS), and heart apex (HA) could be distinguished. In the dorsal surface (view C), the thoracic aorta (TAo), aortic arch (AoAr), brachiocephalic trunk (BT), ascending aorta (AAo), right auricle (RAu), pulmonary veins (PV), pulmonary trunk (PT), coronary sulcus (CS), and left auricle (LAu) were identified. In the cranial aspect (view D), the thoracic aorta (TAo), brachiocephalic trunk (BT), ascending aorta (AAo), caudal vena cava (CaVC), left auricle (LAu), coronary sulcus (CS), right auricle (RAu), pulmonary trunk (PT), left ventricle (LV), right ventricle (RV), paraconal interventricular sulcus (PIS), and heart apex (HA) were visible. In the caudal aspect (view E), the thoracic aorta (TAo), caudal vena cava (CaVC), arteriosus ligament (ArL), pulmonary trunk (PT), aortic arch (AoAr), pulmonary veins (PV), left auricle (LAu), coronary sulcus (CS), left ventricle (LV), and heart apex (HA) were reported.

In the Figure 2, several anatomical structures can be observed: ascending aorta (Aao), aortic valve (AAoV), angular cuspid (AC), arteriosus ligament (ArL), caudal cava vein (CaCV), coronary sulcus (CS), left atrium (LAt), left auricle (LAu), pulmonary trunk (PT), pulmonary valve (PTV), right atrium (RAt), right auricle (RAu), right atrioventricular valve (RAtvV), right ventricle (RV), right ventricle muscle (RVM), right pulmonary artery (RPA), and the left pulmonary artery (LPA).

In Figure 3, the following anatomical components are identified: the ascending aorta (AAo), aortic valve (AoV), right auricle (RAu), right atrioventricular valve (RAtvV), right ventricle (RV), right ventricular muscle (RVM), pulmonary trunk valve (PTV), left ventricle (LV), left auricle (LAu), left atrium (LAt), left atrioventricular valve (LAtvV), pulmonary veins (PV), left atrial appendage (LAAp), fibrous pericardium (FP), right semilunar valve (RSV), septal semilunar valvule (SSV), and left semilunar valve (LSV).

In Figure 4, the following anatomical structures are identified: right atrioventricular valve (RAtvV), septomarginal trabecula (SepmT), right ventricular muscle (RVM), papillary muscle (PM), interventricular septum (IVS), right ventricle (RV), angular cuspid (AngC), parietal cuspid (ParC), left ventricle (LV), septal cuspid (SepC), tendinous cord (TC), left atrioventricular valve (LAtvV), left ventricular muscle (LVM), fibrous pericardium (FP), septal valve (SepV), and parietal valve (ParV).

In Figure 5, the following anatomical structures are identified: right ventricular muscle (RVM), papillary muscle (PM), right ventricle (RV), interventricular septum (IVS), left ventricle (LV), tendinous cord (TC), left ventricular muscle (LVM), and fibrous pericardium (FP).

In Figure 6, the following anatomical structures could be observed: tendinous cord (TC), parietal cuspid (ParC), angular cuspid (AngC), right atrioventricular valve (RAtvV), septomarginal trabecula (SepmT), right ventricle muscle (RVM), papillary muscle (PM), right ventricle (RV), septal cuspid (SepC), right atrium (RAt), heart apex (HA), interventricular septum (IVS), pulmonary trunk (PT), ascending aorta (AAo), right marginal (RM), left ventricle (LV), left atrium (LAt), left atrioventricular valve (LAtvV), left ventricle muscle (LVM), fibrous pericardium (FP), interventricular septum (IAS), pulmonary veins (PV), right atrium (RA), and left atrium (LA).

In Figure 7, several anatomical structures were shown: heart apex (HA), right ventricle muscle (RVM), septomarginal trabecula (SepmT), tendinous cord (TC), papillary muscle (PM), right ventricle (RV), parietal cuspid (ParC), angular cuspid (AngC), right atrium (RAt), right atrioventricular valve (RAtvV), septal cuspid (SepC), interventricular septum (IVS), pulmonary trunk (PT), ascending aorta (AAo), left ventricle (LV), left atrium (LAt), left atrioventricular valve (LAtvV), left ventricle muscle (LVM), right auricle mean diameter (RAMD), interventricular septum (IAS), pulmonary veins (PV), and left auricle mean diameter (LAM).

In Figure 8, several anatomical structures were reported: tendinous cord (TC), parietal cuspid (ParC), angular cuspid (AngC), right atrioventricular valve (RAtvV), septomarginal trabecula (SepmT), right ventricle muscle (RVM), papillary muscle (PM), right ventricle (RV), septal cuspid (SepC), right atrium (RAt), heart apex (HA), interventricular septum (IVS), aortic valve (AoV), ascending aorta (AAo), left ventricle (LV), left atrium (LAt), left atrioventricular valve (LAtvV), left ventricle muscle (LVM), fibrous pericardium (FP), left auricle mean diameter (LAM), mitral valve (MV), and pulmonary trunk (PT).

In Figure 9, several anatomical structures were observed: heart apex (HA), right ventricle muscle (RVM), septomarginal trabecula (SepmT), right ventricle (RV), papillary muscle (PM), interventricular septum (IVS), tendinous cord (TC), parietal cuspid (ParC), angular cuspid (AngC), septal cuspid (SepC), right atrioventricular valve (RAtvV), right atrium (RAt), ascending aorta (AAo), left ventricle (LV), left atrium (LAt), left atrioventricular valve (LAtvV), left ventricle muscle (LVM), fibrous pericardium (FP), aortic valve (AoV), pulmonary veins (PV), interventricular septum (IAS), and left auricle mean diameter (LAM).

Table 2 summarizes the two-dimensional echocardiographic measurements of the evaluated cardiac structures (*N* = 19), reported as median (minimum–maximum). The two-dimensional measurements showed that the left ventricular diastolic diameter (LVd) was the highest recorded value (5.4 cm), followed by the left atrial diastolic diameter (LAd) at 4.2 cm, and the mitral valve diameter (MVD) at 4.5 cm. The systolic thickness of the interventricular septum (IVSs) was 2.3 cm, while the systolic left ventricular free wall (LVFWs) measured 2.7 cm. The smallest dimensions corresponded to the diastolic thickness of the right ventricular free wall (RVFWd) at 1.2 cm and the right ventricular systolic diameter (RVs) at 1.5 cm.

**Table 2:** Representation of the median, minimum, and maximum values of the echocardiographic measurements performed in Shetland ponies (*N* = 19).

Two-dimensional echocardiographic measure	Value (*N* = 19)
IVSs (cm)	2.3 (2.0–3.0)
IVSd (cm)	1.6 (1.2–2.2)
LAd (cm)	4.2 (3.1–5.7)
LAs (cm)	3.3 (1.9–4.9)
RAd (cm)	3.6 (2.6–5.0)
RAs (cm)	1.8 (0.9–2.9)
LVd (cm)	5.4 (4.2–7.3)
LVs (cm)	3.0 (1.9–4.5)
RVd (cm)	3.4 (2.4–4.8)
RVs (cm)	1.5 (0.5–2.7)
LVFWs (cm)	2.7 (1.6–4.0)
LVFWd (cm)	1.8 (1.1–2.6)
RVFWs (cm)	1.8 (1.3–2.4)
RVFWd (cm)	1.2 (0.8–1.7)
TVD (cm)	2.9 (2.2–3.8)
MVD (cm)	4.5 (3.8–5.6)
AoVD (cm)	3.6 (2.7–4.8)
PTVD (cm)	2.7 (2.5–3.1)

Interventricular septum thickness in systole and diástole (IVSs and IVSd); left atrial diameter in diastole and systole (LAd and LAs), right atrial diameter in diastole and systole (RAd and RAs), left ventricular diameter in diastole and systole (LVd and LVs), right ventricular diameter in diastole and systole (RVd and RVs), left ventricular free wall thickness in systole and diastole (LVFWs and LVFWd), right ventricular free wall thickness in systole and diástole (RVFWs and RVFWd), tricuspid valve diameter (TVD), mitral valve diameter (MVD), aortic valve diameter (AoVD), and pulmonary trunk valve diameter (PTVD).

In the 19 ponies included, intra-observer variability was low. For the linear measurements performed, an ICC of 0.94 (95% CI: 0.88–0.98) was obtained, with a mean bias of −0.2 mm and limits of agreement from −1.8 to 1.4 mm in the Bland–Altman analysis. The intra-observer coefficient of variation was 3.2%. The CV% was 5.6%, confirming adequate repeatability given the relatively small size of the animals evaluated.

## 4 Discussion

The breed chosen for this study was the Shetland pony, recognized for its distinctive anatomical and physiological features, particularly in terms of thoracic conformation and body size, when compared with normal-sized horse breeds (6). The Shetland pony has a robust character, and its domestication has been booming, especially in Western countries, where its popularity as companion animals has grown, becoming increasingly popular as pets (4, 5). Likewise, its importance as an experimental animal is remarkable and it is used as an animal model in numerous investigations (7, 13).

The dissection of cardiovascular structures in Shetland pony specimens enabled a detailed evaluation of cardiac morphology and topographic relationships, supporting the interpretation of echocardiographic images. However, it is important to note that these comparisons are conceptual and illustrative in nature. The anatomical specimens did not originate from the same individuals as those undergoing echocardiography, and no quantitative correlation was performed. Therefore, the anatomical figures should be regarded as educational references to aid in the interpretation of standard two-dimensional echocardiographic views, rather than as evidence of direct anatomical-echocardiographic correlation.

In veterinary medicine, the clinical utility of combining anatomical dissection with imaging modalities has been consistently demonstrated. In equine species, anatomical findings have been validated against imaging techniques, such as thoracic ultrasound in live horses (24) and computed tomography angiography with anatomical dissection in neonatal foals (25), highlighting the complementary role of dissection and imaging. Cardiac research in horses has also benefited from this integrative approach: an association between congenital heart disease diagnosed by echocardiography and necropsy findings was confirmed in Arabian foals (26); a clinical case report documented a pseudoaneurysm, periaortic hematoma, pulmonary fistula, and thoracic aortic aneurysm in a Warmblood mare, with necropsy confirming the rupture and fistulation of the aneurysm (27); another case described a primary cardiac hemangiosarcoma, first identified by echocardiography and later confirmed by dissection (28). Furthermore, echocardiography and anatomical dissection have been jointly employed to investigate ischemic heart disease in horses, providing valuable information on the lesions relevant for clinical evaluation (29).

In order to reduce inter-individual variability and facilitate consistent anatomical interpretation, the animals included in this study were all clinically healthy adults, although relatively young when considering the Shetland pony’s potential lifespan, which may exceed 30 years. Both sexes were represented, and efforts were made to maintain similar body proportions across individuals. Although the body weight range was relatively wide (80.1–208.2 kg), the majority of animals fell close to the group median, and thoracic dimensions such as costal arch circumference and total body length showed limited dispersion. This sample composition likely contributed to the relatively low variability observed in the echocardiographic measurements, as supported by the low intra-observer coefficient of variation (3.2%) and high intraclass correlation coefficient (0.94). These factors support the internal consistency of our findings while still reflecting a representative adult Shetland pony population.

The measurements obtained in the group of Shetland ponies showed lower values than those reported for larger horse breeds (30), including the costal arch (118.5 ± 27.8 vs. 199.0 ± 7.0 cm), total length (97.3 ± 26.1 vs. 188.0 ± 10.0 cm), height at the withers (91.5 ± 17.4 vs. 162.0 ± 4.0 cm), and estimated weight (119.6 ± 62.7 vs. 627.0 ± 59.0 kg), reflecting their smaller physical size. In the echocardiographic study, reference images and measurements of different cardiac structures were obtained, as well as heart rate, calculated between two consecutive beats, with an average of 53.4 ± 18.7 bpm. This value was higher than that reported in a previous study on pony mares (40.4 ± 13.3 bpm) (31). Although heart rate variability has been considered as potentially related to stress, physiological discomfort, or seasonal influences, in this study the animals were examined in a calm standing position, in their usual environment, without sedation and with minimal restraint. An adaptation period was allowed prior to echocardiography to minimize handling-related excitation, and during the procedure the operator systematically monitored for clinical signs of stress or discomfort (e.g., ear position, restlessness, defensive movements, respiratory pattern). No overt signs of distress were observed, suggesting that the higher heart rate values recorded are more likely attributable to individual variability and seasonal or environmental factors rather than acute stress during the examination.

In equine medicine, right-sided echocardiographic views are widely employed and represent the standard approach for cardiac evaluation. However, as previously reported by Koenig et al. (20), obtaining left apical views in adult horses is often not feasible due to the deep intrathoracic position of the heart and the interposition of thoracic structures. In contrast, McConachie et al. (32) noted improved feasibility in foals and animals with smaller thoraxes. In our study, left-sided apical views were successfully obtained in all Shetland ponies; however, the clearest and most anatomically defined images were consistently observed in younger animals with lower body size and weight, most of whom were females. This suggests that while these views are feasible in this breed, their quality and reproducibility may be influenced by age, sex, and thoracic conformation, factors that should be considered when extrapolating to other populations or clinical contexts.

Only a limited number of studies have reported two-dimensional echocardiographic values in Shetland ponies, mostly based on small sample sizes. The present measurements contribute additional structural data and may serve as a foundation for future studies. Although functional assessment is clinically relevant, it was not included here due to methodological constraints, as it requires additional echocardiographic techniques beyond the scope of this descriptive work. Findings on left ventricular free wall thickness (LVFW) in clinically healthy Shetland ponies are consistent with those of D’Fonseca et al. (11), who reported a significant increase in mean left ventricular wall thickness in female ponies subjected to a long-term high-energy diet. This concordance supports the relevance of LVFW as a reliable anatomical parameter sensitive to hypertrophic changes in this breed. Although Shetland ponies are predisposed to hypertension, obesity, and secondary ventricular hypertrophy according to previous literature, our study did not include blood pressure measurements, and no evidence of overt left ventricular hypertrophy was observed in the examined animals. The animals analyzed presented body condition scores comparable to those previously reported for this breed, which may reflect their tendency to overweight, but within the limits compatible with clinically healthy status.

In comparison with the echocardiographic values reported by Sleeper et al. (13) in Shetland ponies, the present study revealed similar interventricular septal and free wall thicknesses, but smaller left ventricular and aortic root diameters. These differences may be explained by the characteristics of the study populations: while Sleeper et al. (13) examined only stallions with relatively high body condition scores, the present cohort included both males and females of varying age and body size. Differences in sex distribution and body condition are therefore likely to influence cardiac dimensions and account for the discrepancies observed between studies. Furthermore, when considering the allometric reference models proposed by Al-Haidar et al. (17) across multiple equine breeds and ponies (not exclusively Shetland ponies), the present values fall within the expected ranges for animals of comparable body size, reinforcing their suitability as breed-relevant measurements.

In contrast, multiple studies have reported two-dimensional mode echocardiographic parameters for larger equine breeds, where values are generally similar across Standard, Warmblood, and Friesian horses (17, 30, 33). In Shetland ponies, however, notably smaller cardiovascular structures were observed. For instance, the left ventricular diameter was approximately half that recorded in larger breeds. Reported IVSs and IVSd values in Standard horses are 4.6 and 3.1 cm, compared to 2.5 and 1.7 cm in this study. Similarly, LVs and LVd values in Standard (7.0 and 10.7 cm), Warmblood (11.6 and 17.0 cm), and Friesian horses (10.8 and 15.9 cm) (30, 33) far exceed those in Shetland ponies (3.2 and 5.7 cm). These differences indicate that left ventricular internal diameters in large-breed horses are more than twice those measured in the ponies. In contrast, LVFW and LVFWd values showed smaller differences between breeds, with Standard horses measuring 3.9 and 2.7 cm and Shetland ponies 2.8 and 1.9 cm. Vascular measurements have also been previously reported, for example the diameter of the Aorta for the standard horse was 7.2 (33), being a value similar to those reported for purebred animals. In the previous case, the value is higher than those of the Shetland pony group, in which a value of 3.8 was shown.

When compared with the echocardiographic values reported by Farag et al. (34) in healthy donkeys, Shetland ponies exhibited smaller cardiac dimensions overall. For example, left ventricular diameters were lower in ponies (LVIDd 5.4 cm; LVIDs 3.0 cm) than in donkeys (6.1 cm and 3.7 cm, respectively). Similarly, left ventricular free wall thicknesses were reduced in ponies (1.8 and 2.7 cm in diastole and systole) compared with donkeys (2.8 and 3.6 cm). Interventricular septal thickness in diastole was comparable between species (1.6 vs. 1.7 cm), but donkeys showed greater systolic values (2.9 vs. 2.3 cm). These differences are most likely attributable to the greater body size of donkeys, while Shetland ponies display proportionally more compact ventricular dimensions.

While echocardiographic dimensions in Shetland ponies showed similarities to those reported in other small equine breeds, notable differences were observed when compared to large-breed horses. These differences may have clinical consequences, as applying reference values from other breeds could lead to misclassification of cardiac dimensions or incorrect interpretation of normal variants as pathological. Therefore, establishing breed-specific reference data is important to improve diagnostic accuracy and guide appropriate clinical decision-making in Shetland ponies.

The results obtained in this study are subject to several important limitations. First, the sample size was relatively small and geographically restricted, which did not allow for a formal sample size calculation and may limit the generalizability of the findings to the broader Shetland pony population. Furthermore, this was an exploratory and descriptive study, designed to provide preliminary anatomical and echocardiographic reference information rather than to establish definitive normative values. The study population consisted predominantly of young Shetland ponies, which may have influenced the echocardiographic measurements and morphological observations reported. In equines, cardiac dimensions can change with age due to physiological remodeling, altered loading conditions, or subclinical degenerative processes. As such, the present findings should be interpreted primarily as reference values for young, healthy individuals, and may not fully capture variations present in older ponies. Future studies incorporating a broader age range would be valuable to define age-specific reference intervals and assess potential remodeling patterns across the lifespan.

Inter-observer reproducibility was not analyzed, as all measurements were performed by a single experienced operator. Furthermore, reproducibility was evaluated using a pooled ICC, without individual assessment for each parameter. While this approach indicates overall consistency, it may limit the extrapolation of the results to other evaluators or clinical settings, particularly for technically complex measurements. All ultrasound studies were performed with the same ultrasound machine. None of the anatomical images can be considered a perfect reference for the echocardiographic views; in particular, the transverse view optimized for visualization of the pulmonary trunk bifurcation (Figure 2) showed the poorest correspondence, mainly due to the absence of an extended segment of pulmonary arterial vasculature in the available specimens. In addition, adequate visualization of the mitral valve (Figure 4) could not be achieved, further limiting the anatomical echocardiographic correspondence. It should be noted that no direct caliper measurements of septal or wall thickness were performed on the anatomical specimens, since tissue freezing and sectioning may cause distortion of dimensions. Therefore, the study was not intended to establish precise numerical correlations with echocardiographic measurements, but rather to provide a visual anatomical reference for the interpretation of echocardiographic views in Shetland ponies.

In short, the present study aims to be an initial reference for the subsequent extension of the investigation of the echocardiographic and macroscopic measurements of the Shetland pony, in addition to serving as a visual atlas for the identification of the most important cardiovascular structures in echocardiography.

## 5 Conclusion

This study provides a detailed description of the main cardiovascular structures identifiable through two-dimensional echocardiography in healthy Shetland ponies, and offers anatomical reference images to support the interpretation of these structures in a breed-specific context. The echocardiographic measurements obtained were, as expected given the smaller body size of Shetland ponies, generally lower than those reported for larger equine breeds. These findings add complementary data to the limited information currently available for this breed and may contribute to future efforts aimed at refining breed-specific cardiovascular parameters.

## Data Availability

The original contributions presented in the study are included in the article/supplementary material, further inquiries can be directed to the corresponding authors.

## Glossary

AAo: Ascending aorta
AngC: Angular cuspid
AoAr: Aortic arch
AoV: Aortic valve
AoVD: Aortic valve diameter
ArL: Arteriosus ligament
BCS: Body condition score
BT: Brachiocephalic trunk
Bpm: Beats per minute
CA: Costal arch circumference
CrVC: Cranial vena cava
CS: Coronary sulcus
CaVC: Caudal vena cava
FP: Fibrous pericardium
HA: Heart apex
IAS: Interatrial septum
IVS: Interventricular septum
IVSd: Interventricular septum thickness in diastole
IVSs: Interventricular septum thickness in systole
LA: Left atrium
LAAp: Left atrial appendage
LAd: Left atrial diameter in diastole
LAu: Left auricle
LAM: Left auricle mean diameter
LAs: Left atrial diameter in systole
LAt: Left atrium
LAtvV: Left atrioventricular valve
LPA: Left pulmonary artery
LV: Left ventricle
LVFWd: Left ventricular free wall thickness in diastole
LVFWs: Left ventricular free wall thickness in systole
LVM: Left ventricular muscle
LVd: Left ventricular diameter in diastole
LVs: Left ventricular diameter in systole
MV: Mitral valve
MVD: Mitral valve diameter
PM: Papillary muscle
ParC: Parietal cuspid
ParV: Parietal valvule
PIS: Paraconal interventricular sulcus
PT: Pulmonary trunk
PTV: Pulmonary trunk valve
PTVD: Pulmonary trunk valve diameter
PV: Pulmonary veins
RA: Right atrium
RAd: Right atrial diameter in diastole
RAs: Right atrial diameter in systole
RAtvV: Right atrioventricular valve
RAu: Right auricle
RAMD: Right auricle mean diameter
RM: Right marginal
RSV: Right semilunar valve
RPA: Right pulmonary artery
RV: Right ventricle
RVFWd: Right ventricular free wall thickness in diastole
RVFWs: Right ventricular free wall thickness in systole
RVM: Right ventricular muscle
RVd: Right ventricular diameter in diastole
RVs: Right ventricular diameter in systole
SepC: Septal cuspid
SepV: Septal valve
SepmT: Septomarginal trabecula
SIS: Subsinuosal interventricular sulcus
SSV: Septal semilunar valve
TAo: Thoracic aorta
TC: Tendinous cord
TL: Total body length
TVD: Tricuspid valve diameter
